# Regulation of Aryl Hydrocarbon Receptor Interacting Protein (AIP) Protein Expression by MiR-34a in Sporadic Somatotropinomas

**DOI:** 10.1371/journal.pone.0117107

**Published:** 2015-02-06

**Authors:** Judit Dénes, Leandro Kasuki, Giampaolo Trivellin, Leandro M. Colli, Christina M. Takiya, Craig E. Stiles, Sayka Barry, Margaret de Castro, Mônica R. Gadelha, Márta Korbonits

**Affiliations:** 1 Department of Endocrinology, William Harvey Research Institute, Barts and the London School of Medicine, Queen Mary University of London, London, United Kingdom; 2 Semmelweis University, School of PhD studies, Doctoral School of Clinical Medicine, Budapest, Hungary; 3 Endocrinology Unit, Clementino Fraga Filho University Hospital, Federal University of Rio de Janeiro, Rio de Janeiro, Brazil; 4 Department of Internal Medicine, Endocrinology Laboratory, Ribeirão Preto Medical School, São Paulo University, São Paulo, Brazil; 5 Biofísica Carlos Chagas Filho Institute, Federal University of Rio de Janeiro, Rio de Janeiro, Brazil; University of Cordoba, SPAIN

## Abstract

**Introduction:**

Patients with germline *AIP* mutations or low AIP protein expression have large, invasive somatotroph adenomas and poor response to somatostatin analogues (SSA).

**Methods:**

To study the mechanism of low AIP protein expression 31 sporadic somatotropinomas with low (n = 13) or high (n = 18) AIP protein expression were analyzed for expression of *AIP* messenger RNA (mRNA) and 11 microRNAs (miRNAs) predicted to bind the 3’UTR of *AIP*. Luciferase reporter assays of wild-type and deletion constructs of *AIP*-3’UTR were used to study the effect of the selected miRNAs in GH3 cells. Endogenous AIP protein and mRNA levels were measured after miRNA over- and underexpression in HEK293 and GH3 cells.

**Results:**

No significant difference was observed in *AIP* mRNA expression between tumors with low or high AIP protein expression suggesting post-transcriptional regulation. miR-34a was highly expressed in low AIP protein samples compared high AIP protein adenomas and miR-34a levels were inversely correlated with response to SSA therapy. miR-34a inhibited the luciferase-*AIP*-3’UTR construct, suggesting that miR-34a binds to *AIP*-3’UTR. Deletion mutants of the 3 different predicted binding sites in *AIP*-3’UTR identified the c.*6–30 site to be involved in miR-34a’s activity. miR-34a overexpression in HEK293 and GH3 cells resulted in inhibition of endogenous AIP protein expression.

**Conclusion:**

Low AIP protein expression is associated with high miR-34a expression. miR-34a can down-regulate AIP-protein but not RNA expression *in vitro*. miR-34a is a negative regulator of AIP-protein expression and could be responsible for the low AIP expression observed in somatotropinomas with an invasive phenotype and resistance to SSA.

## Introduction

Germline mutations have been described in the *aryl hydrocarbon receptor interacting protein (AIP)* gene in the setting of either familial isolated pituitary adenoma (FIPA) or in simplex, young-onset, predominantly growth hormone (GH)- and/or prolactin-secreting pituitary adenomas [1, 2]. *AIP* is a tumor suppressor gene and patients with acromegaly harboring germline mutations in this gene have more invasive tumors showing sparsely-granulated pattern and are less likely to respond to treatment with somatostatin analogues (SSA) [3–5].

Although no somatic *AIP* mutation has been described in pituitary adenomas to date [4, 6], approximately half of the sporadic somatotropinomas present with low intrinsic AIP expression, and, similar to the mutated tumors, most of them are invasive and respond poorly to SSA therapy [7–9]. Therefore, low AIP expression seems to be important in determining the pathological characteristics of somatotropinomas.

MicroRNAs (miRNAs) are small non-coding RNAs with an important role in post-transcriptional regulation of mRNA and/or protein expression. miRNAs were found to be involved in the pathogenesis of many human diseases, including pituitary adenoma [10–14]. We hypothesized that miRNA regulation of AIP protein expression could be responsible for the low AIP levels found in approximately half of the sporadic somatotropinomas [8, 9].

## Subjects and Methods

### Patients

Thirty-four consecutive patients with acromegaly who had previously had pituitary surgery and had tissue available (paraffin block and fresh frozen tumor sample) were included in the study. This study was approved by the Ethics Committees of the Clementino Fraga Filho University Hospital/Medical School, Federal University of Rio de Janeiro and the Clinics Hospital, Ribeirão Preto Medical School, São Paulo University. All subjects gave written informed consent before study entry. Patients underwent pituitary surgery between 2006 and 2011. Biochemical diagnosis of acromegaly was based on international criteria [15, 16]. Exclusion criteria included previous known *AIP* mutations, a family history of pituitary adenoma, presence of features or family history of Carney complex or multiple endocrine neoplasia type 1 or 4 and preoperative therapy with SSA [as treatment may increase AIP expression [17]]. Tumor invasiveness was determined according to Knosp-Steiner criteria [18]. GH-secreting pituitary tumor samples were obtained during transsphenoidal surgery: part of the sample was processed for routine histopathological and immunohistochemical studies (including anterior pituitary hormones), and part was snap-frozen and stored at -70°C for molecular biology studies. All samples were micro-dissected by an experienced pathologist in order to separate any non-tumoral tissue and homogenized using a Polytron homogenizer. In addition, five normal human pituitaries were obtained within 10 hours from the time of death at autopsies of subjects who had died from natural causes without previous evidence of any endocrine disease or pituitary abnormality.

### Postsurgical evaluation

Biochemical assessment was performed 12 weeks after surgery by evaluation of oral glucose tolerance test (OGTT) and serum insulin-like growth factor I (IGF-I) levels in all subjects. Pituitary magnetic resonance imaging (MRI) was performed 3 months after the surgical procedure. Patients were considered as non-cured on the basis of the clinical picture, nadir GH levels after OGTT higher than 0.4 ng/mL, and plasma IGF-I levels higher than age-matched normal subjects. Medical therapy with long-acting octreotide (OCT-LAR) was started at a dose of 20 mg every 4 weeks, and the dose was increased to 30 mg every 4 weeks in uncontrolled patients after 3 months of therapy. Efficacy of medical therapy was evaluated at the last patient visit, and patients were considered uncontrolled if they had a basal GH value higher than 1.0 ng/mL and/or a plasma IGF-I level higher than age-matched normal subjects with at least 6 months of treatment with OCT-LAR at a dosage of 30 mg. Postsurgical follow-up ranged from 12 to 60 months (median 32 months).

Tumor volume was not considered as an endpoint in this series because the study included only postsurgical patients, which could lead to mistakes in the volume measurements due to confounding variables such as postsurgical changes.

### Cytokeratin pattern analysis

The cytokeratin expression pattern was analyzed as previously published with a mouse monoclonal antibody CAM 5.2 (1:100, BD Biosciences, San Jose, CA, USA, cat. number 349205) [19]. Tumors were classified according to the cytokeratin expression as densely granulated, sparsely granulated or mixed forms according to a previously reported classification [20]. Mixed tumors were considered as densely granulated for analysis, as previously suggested [20].

### *AIP* mutation analysis in somatotropinomas

Deoxyribonucleic acid (DNA) was extracted using the QIAamp DNA Mini Kit (Qiagen, Valencia, CA, USA) from the pituitary adenoma tissue according to the manufacturer’s protocol. The entire coding sequence of *AIP* (NM_003977.2), the conserved splice sites (from the conserved A of the upstream branch site to +10 downstream of each exon) and 1200 base pairs of the promoter region were direct sequenced, as previously published [4]. For those tumors whose DNA was not available, the complementary DNA (cDNA) was sequenced with previously published primers [4]. Sequencing was performed with ABI 3130 Genetic Analyzer (ABI PRISM/PE Biosystems, Foster City, CA, USA).

### AIP mRNA and protein expression analysis in somatotropinomas

**Immunohistochemistry.** AIP expression was analyzed by immunohistochemistry, using a monoclonal antibody (1:500, NB100–127, Novus, Littleton, CO, USA) in paraffin-embedded tissue sections as previously described [7, 8]. For semi-quantitative estimate of cytoplasmic AIP immunostaining, slides were scored for pattern [diffuse (score 2) or patchy (score 1)] and for intensity [strong (score 3), moderate (score 2) and weak (score 1)], and the final score was calculated by multiplying the two scores (pattern and intensity), as previously described [7, 8]. Final scores of 0 (no expression), 1 and 2 were considered as low AIP expression, while scores 3, 4, or 6 were considered as high expression. The adenoma scoring was performed by a single independent observer (L.K.) blinded for the clinical data of the patients.

**Reverse Transcription-qPCR.** The AIP mRNA expression was analyzed by real-time qPCR in somatotropinomas and normal pituitaries. Tumoral ribonucleic acid (RNA) was extracted using the RNeasy Mini kit (Qiagen) according to the manufacturer’s protocol. The amount and quality of the extracted RNA were evaluated using NanoDrop 2000 (Thermo Fischer, Wilmington, DE, USA). Approximately one microgram of total RNA was used in a reverse transcription reaction of 10 μL using 2.5 μM Oligo D(T), 5.5 mM MgCl_2_, 2.0 mM dNTPs, 20 U/μL RNase Inhibitor, 50 U/μL MultiScribe TaqMan and 10x RT Buffer, in a first strand cDNA synthesis kit (Taq-Man RT reagents, Applied Biosystems, Branchburg, New Jersey, USA). The reverse transcription cycle sequence was 25°C for 10 min, 48°C for 30 min and 95°C for 5 min. The cDNA of *AIP* and of *glucuronidase β* (*GUSB*), *TATA box binding protein* (*TBP*) and *phosphoglycerate kinase 1* (*PGK1*) genes, used as endogenous controls, were separately amplified in duplicates, in a total volume of 12 μl, in real-time qPCR assays, using the Applied Biosystems 7500 Real-Time PCR System (Foster City, CA, USA). Reactions were incubated in a 96-well optical plate at 95°C for 10 min, followed by 40 cycles of 95°C for 15 sec and 60°C for 1 min. The TaqMan assays for *AIP* and the endogenous controls are shown in Table 1. The cycle threshold (Ct) was defined as the cycle number at which the fluorescence surpasses the fixed threshold. The Ct data were performed using default threshold settings. Expression analysis was performed with the QPCR software [21]. Efficiency of each reaction was calculated by linear regression with the LingRegPCR software. The normalization of each sample results was performed by subtracting the Ct (geometric mean) for the target gene (*AIP*) by the endogenous control Ct (*TBP*, *PGK1* and *GUS*), generating the ΔCt [Ct_sample_ (target gene)—Ct_sample_ (endogenous control)]. The normalized results (ΔCt) were then subjected to calibration step. We used five normal pituitary tissue samples as calibrator, obtaining the ΔΔCt (ΔCt_sample_- ΔCt_normal pituitary_). The relative expression of each gene was given by the formula (1 + efficiency)^-ΔΔCt^. The efficiency value was calculated for each reaction. The median values​​ obtained from (1 + efficiency)^-ΔΔCt^ of tumor samples was compared with the median value of (1 + efficiency)^-ΔΔCt^ normal pituitary tissue samples, obtaining the fold change. Adequacy of endogenous controls was calculated with the GeNorm 3.3 visual basic application for Microsoft Excel.

**Table 1:** TaqMan assays used in real-time qPCR quantification.

Genes and miRNAs	Assays (TaqMan Applied Biosystems)
human AIP	Hs_00610222_m1
rat Aip	Rn_00597273-m1
hsa-let-7a	000377
hsa-let-7b	002619
hsa-miR-202	002363
hsa-miR-22	000398
hsa-miR-34a	000426
hsa-miR-34c	000428
hsa-miR-324	002161
hsa-miR-449	001030
hsa-miR-510	002241
hsa-miR-612	001579
hsa-miR-639	001583
hsa-miR-671	002322
Endogenous controls	
RNU38B	001004
RNU49	001005
RNU6B	4373381
Beta-actin	4352340E
TBP	Hs_00427621_m1
GUS Beta	Hs_00939627_m1
PGK1	Hs_99999906_m1
GAPDH	Hs_99999905_m1

### Identification and expression analysis of miRNAs targeting *AIP* 3’-untranslated region (3’-UTR) in somatotropinomas

**Target site prediction.** To identify AIP mRNA-miRNA interaction we initially used algorithms described in the miRNAmap prediction program [22]. This bioinformatics tool uses data from TargetScan 6.0 (http://www.targetscan.org), MiRanda (http://www.microrna.org/microrna/home.do) and RNAhybrid (http://bibiserv.techfak.uni-bielefeld.de/rnahybrid/). The mRNA-miRNA interaction was evaluated by three different criteria: (i) target site predicted by at least two prediction programs, (ii) the target gene contains multiple target sites for the miRNA and (iii) the target sites are located in accessible regions of the RNA as determined by a pre-specified algorithm. In addition, miRNAmap used miRNA and target mRNA expression profiles from a repository to calculate the Pearson correlation coefficients for each miRNA and the target gene [22, 23]. We selected to evaluate by qPCR miRNAs that reach all three miRNAmap criteria or at least two miRNAmap criteria and a negative Pearson coefficient at least -0.30. Moreover, in order to confirm and to complete our search for miRNAs interacting with *AIP* and to determine the exact miRNA target binding sites in both human and rat *AIP* we utilized TargetScan version 6.2 (http://www.targetscan.org), MicroCosm (http://www.ebi.ac.uk/enright-srv/microcosm/cgi-bin/targets/v5), FindTar version 3 (http://bio.sz.tsinghua.edu.cn/content/list/), miRanda (http://www.microrna.org/microrna/home.do) and PicTar (http://www.pictar.bio.nyu.edu).

**Real-time qPCR quantification of miRNAs identified by in silico target prediction.** The selected miRNA expressions were analyzed by real-time qPCR in somatotropinomas and in normal pituitaries. The reverse transcription cycle for miRNAs was 16°C for 30 min, 42°C for 30 min and 85°C for 5 min. The cDNA of the selected miRNAs and of *RNU38B* and *RNU49*, used as endogenous controls, were amplified in duplicates in three different reactions in a total volume of 12 μl, in real-time qPCR assays, using the Applied Biosystems 7500 Real-Time PCR System. Reactions were incubated in a 96-well optical plate with 40 cycles of 95°C for 15 sec and 60°C for 1 min. The TaqMan assays for miRNAs and for the endogenous controls are shown in Table 1. The analysis of the results was performed with the same methodology previously described for *AIP*.

### Cell culture

The rat GH- and prolactin-secreting pituitary adenoma cell line GH3 [24, 25] and the human embryonic kidney cell line HEK293 [26] were grown in Dulbecco’s modified Eagle’s medium (DMEM, Sigma-Aldrich, Poole, Dorset, UK) supplemented with 10% fetal bovine serum (Biosera, Ringmer, UK), penicillin (100 IU/mL) and streptomycin (100 mg/mL, Sigma Aldrich) in a humidified atmosphere at 37^o^C with 5% CO_2_. Cells were obtained from the European Collection of Cell Cultures.

### Generation of mutant *AIP*-3’-UTR Reporter Plasmids

A pGL3-vector containing the human *AIP*-3’-UTR was used to perform the experiments [27]. A 931-bp segment of human *AIP*-3’-UTR is located immediately downstream from the coding sequence of the *Firefly* luciferase reporter gene. To examine whether the effect on the luciferase activity of the studied miRNAs was specifically due to binding to the predicted binding sites in the *AIP*-3’-UTR fragment, we disrupted these sites by site-directed mutagenesis. For interrupting the perfect “seed” pairing, four nucleotides (miR-34a site A and B) or three nucleotides (site C) of the miR-34a seed sequences were deleted using the QuikChange XL-site-directed mutagenesis kit (Agilent Technologies, Santa Clara, CA, USA) and the following primers: site A: forward 5'-ggccctgccttaccaagcccactgct-3' and reverse 5'-agcagtgggcttggtaaggcagggcc-3', site B: forward 5'-cctgccaagcccctgcagctgcca-3' and reverse 5'-tggcagctgcaggggcttggcagg-3', site C: forward 5'-gcccactgctgcccagcccccctg-3' and reverse 5'-caggggggctgggcagcagtgggc-3'. Three mutant plasmids were generated with deletions at site A (MUT_A), B (MUT_B) and C (MUT_C), and a further mutant was generated with both site A and C mutations. All mutant inserts were confirmed by direct sequencing.

### Luciferase gene reporter assay

GH3 cells were seeded in the inner wells of 24-well plates [28] at a density of 1x10^5^ cells/well. After 24h, cells were co-transfected using Lipofectamine 2000 (Invitrogen, Paisley, UK) with 0.5 μg of the pGL3-vector and 25 ng of the Renilla vector [pRL-cyto megalovirus (CMV)] as previously described [27]. For each plate, the pre-miR-34a (PM11030, Life Technologies) or pre-miR-22 (PM11752, Life Technologies) or the scrambled pre-miR (AM17111, Life Technologies) was co-transfected at a final concentration of 50 nM. *Firefly* and *Renilla* luciferase activities were measured consecutively 24h post-transfection using the Dual-Luciferase Reporter Assay System (Promega, Southampton, UK) as previously described [27]. Ratios of *Firefly* vs. *Renilla* luminescence signals served as a measure for reporter activity normalized for transfection efficiency.

### Endogenous miR-34a expression in different cell lines and tissues

In order to estimate the level of expression of miR-34a in the GH3 and HEK293 cells we extracted RNA from these cells using the RNeasy Mini Kit (Qiagen). We also included in the analysis RNA from human tissues (AM6000, Ambion) previously described to express miR-34a at high (ovary, prostate and testes) or low (adipose, heart and liver) levels (http://mirnamap.mbc.nctu.edu.tw). Real-time qPCR amplifications were run using the hsa-miR-34a TaqMan MicroRNA Assay, (4427975, Life Technologies). RNU6B was used as an endogenous control for human samples and beta-actin was chosen as a control for the rat sample. The analysis of the results was performed with the same methodology previously described for the *AIP* qPCR.

### Endogenous AIP mRNA and protein expression after miR-34a overexpression and inhibition

HEK293 and GH3 cells were seeded in 24-well plates at a density of 0.6x10^5^ cells/well and 1x10^5^ cells/well, respectively. After 1–24 h cells were transfected with the pre-miR-34a precursor, the anti-miR-34a inhibitor (AM11030, Life Technologies), scrambled-miR or scrambled-anti-miR (AM17010, Life Technologies) at a final concentration of 50 nM. Twenty-four and forty-eight hours later cells were harvested and proteins and RNA extracted. RT-qPCR was performed with the TaqMan system using ready made AIP-probe primer kits (Hs_00610222_m1, Rn_00597273-m1, Life Technologies). Reactions were performed in triplicate using glyceraldehyde 3-phosphate dehydrogenase (*GAPDH)* as endogenous control. Data were analyzed as previously described [27]. Protein extraction and quantification were performed as described previously [29]. Twenty to 80 μg of cell culture lysates were separated by electrophoresis and transferred onto nitrocellulose membranes. Membranes were incubated with mouse monoclonal AIP antibody (Novus NB100–127) at 1:1000 dilution and GAPDH rabbit antibody (sc-25778 Santa Cruz Biotechnology, Dallas, USA) at 1:1000 dilution was used as loading control. Infrared fluorescent-labeled anti-rabbit or anti-mouse secondary antibodies (IRDye 680 and 800, Li-Cor Biosciences, Cambridge, UK) were used at a 1:8000 dilution. Immunoblot detection and density measurements were performed on the Odyssey infrared-imaging system (Li-Cor).

### Statistical analysis

The statistical analysis was performed using SPSS version 16.0 for Windows (SPSS, Inc., Chicago, IL, USA) or StatsDirect software (Addison-Wesley-Longman, Cambridge, UK). The results were reported as median values (minimum—maximum) or mean ± SEM of two to ten independent experiments, each performed in triplicate. The Student t-test or the Mann-Whitney test was used as appropriate to compare numerical variables. The chi-squared test was used to compare categorical variables. *P* values < 0.05 were considered statistically significant.

## Results

### Analysis of *AIP* mutations

A total of 34 tumors from acromegalic patients were selected for the study. Tumor genomic DNA (gDNA) from 28 patients and tumor cDNA from six patients were sequenced for *AIP* mutations. Two patients were identified with truncating *AIP* mutations (p.Y268*, and p.R304*) and one patient with a variant with controversial significance (p.R16H) [30–32]. Leukocyte-derived DNA from these patients confirmed heterozygous germline mutations and these three patients were excluded from the study; therefore only data from 31 adenomas were included in following experiments.

### Demographic, radiological, biochemical and pathological data of the patients with acromegaly

The demographic, biochemical and pathological data of the 31 patients included in the study are summarized in Table 2. The median age at diagnosis was 43 years (range 23–63), 15 patients (48%) were male. The median GH at diagnosis was 23.0 ng/mL (range 1.6–392.5) and the median IGF-I was 408% of the upper limit of normal range (range 165–1139). Twenty-seven tumors (87%) were macroadenomas and 15 (48%) co-expressed GH and prolactin upon immunostaining.

**Table 2:** Demographic, radiological, biochemical and pathological characteristics of the patients with acromegaly.

Patient	Age at diagnosis (years)	Sex	Tumor Size (cm)	Invasive*	Baseline GH (ng/mL)	Baseline IGF-I (%ULNR)	Control with OCT-LAR	AIP protein expression (score)	miR-34a#	Granulation pattern	PRL
1	52	M	3.8 x 3.5	Y	251.0	331	N	L (2)	1.50	Sparsely	Y
2	31	F	2.1 x 1.9	N	53.9	302	N	H (3)	1.37	Sparsely	N
3	55	M	4.0 x 3.7	Y	9.2	380	Y	L (1)	0.66	N/A	N
4	46	M	3.0 x 2.1	Y	51.5	417	N	H (3)	1.30	Densely	Y
5	37	M	3.3 x 3.0	Y	34.2	401	NT	L (2)	0.73	Sparsely	Y
6	63	F	3.0 x 2.5	Y	133.0	225	N	H (4)	0.50	Densely	N
7	40	F	3.0 x 2.8	Y	4.3	372	N	L (2)	1.40	Densely	N
8	46	M	2.6 x 2.6	Y	15.6	208	Y	H (6)	0.13	Densely	Y
9	23	F	2.0 x 1.5	Y	110.0	165	N	H (4)	0.65	N/A	N
10	63	M	1.0 x 0.8	N	3.6	386	NT	H (4)	2.50	N/A	N
11	46	F	3.4 x 2.3	Y	53.5	415	N	L (2)	2.27	Sparsely	N
12	42	M	1.1 x 0.7	N	9.5	488	N	H (4)	0.79	Densely	Y
13	43	F	1.0 x 0.8	N	15.5	238	Y	H (4)	0.13	N/A	Y
14	42	M	1.5 x 1.3	Y	23.0	1139	NT	L (2)	0.57	Sparsely	Y
15	59	F	1.0 x 0.9	N	1.7	805	Y	H (4)	0.11	Densely	Y
16	34	M	1.2 x 1.0	N	7.1	199	Y	H (4)	0.08	Densely	Y
17	50	F	1.2 x 1.0	N	39.2	NA	Y	H (6)	0.15	Densely	N
18	30	F	1.3 x 2.0	N	13.7	642	Y	H (3)	0.16	Densely	N
19	37	F	3.0 x 2.5	N	185.0	531	Y	H (4)	4.96	Densely	N
20	36	M	1.5 x 1.0	N	70.0	NA	N	H (4)	1.11	Densely	N
21	57	M	2.0 X 1.4	Y	47.8	963	N	L (2)	0.28	Sparsely	N
22	52	F	3.3 x 3.4	Y	23.0	517	N	L (2)	2.64	Sparsely	Y
23	32	M	3.8 x 2.6	Y	10.8	NA	N	H (6)	1.69	Sparsely	Y
24	54	F	2.3 x 1.7	Y	32.5	490	N	L (2)	2.42	Sparsely	N
25	56	F	1.8 x 1.3	Y	1.6	587	N	L (2)	1.81	Densely	N
26	53	F	2.5 x 1.8	N	20.9	NA	N	H (3)	0.32	Sparsely	N
27	43	M	0.9 x 0.7	N	4.9	789	NT	L (2)	1.82	N/A	N
28	37	F	2.9 x 1.9	Y	10.0	265	NT	L (1)	3.37	Sparsely	Y
29	42	M	1.8 x 1.5	N	110.0	NA	Y	H (6)	0.61	Densely	Y
30	31	F	1.3 x 1.1	N	119.0	NA	N	L (2)	0.32	Densely	Y
31	24	M	5.8 x 5.0	Y	392.5	NA	Y	H (4)	0.12	N/A	Y

F, female

M, male

Y, yes

N, no

NA, not available

ULNR, upper limit of normal range

L, low

H, high

AIP, aryl hydrocarbon receptor interacting protein

OCT-LAR, octreotide LAR

NT, not treated

*, tumor invasiveness was determined according to Knosp-Steiner criteria

^#^, expression level (fold change)

NT, not treated with somatostatin analogues, PRL, immunostaining for prolactin.

### AIP protein levels and correlation with *AIP* mRNA levels

**Fig 1:**
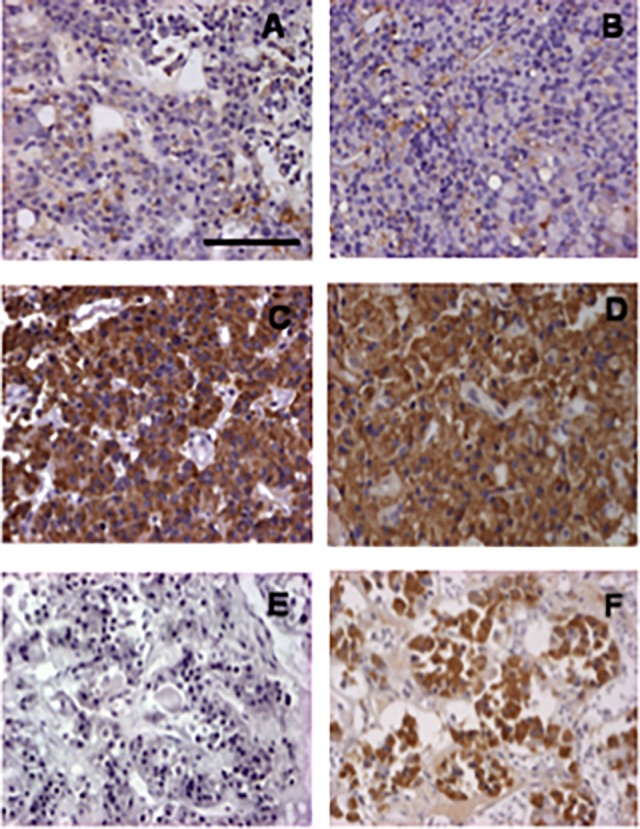
Aryl hydrocarbon receptor interacting protein (AIP) immunostaining. A and B—Examples of low AIP expression; C and D: Examples of high AIP expression; E—Normal human pituitary staining with omitting primary antibody (negative control); F—Normal human pituitary staining with AIP (positive control); Scale bar = 1000 μm.

All tumors expressed AIP, with low expression levels (score 1–2) observed in 13 cases (42%) (Fig. 1). Interestingly, there was no difference in the *AIP* mRNA expression between tumors with low or high AIP protein levels: in the low AIP protein group the median *AIP* mRNA expression was 0.91 (range 0.48–1.95) and in the high AIP protein group was 1.14 (0.45–2.34, low vs. high protein group *P =* 0.123). These data lead us to the hypothesis that post-transcriptional regulation, such as that exerted by miRNAs, may be the cause of the low AIP protein expression.

### miRNA expression levels in patients with low or high AIP protein expression

**Fig 2:**
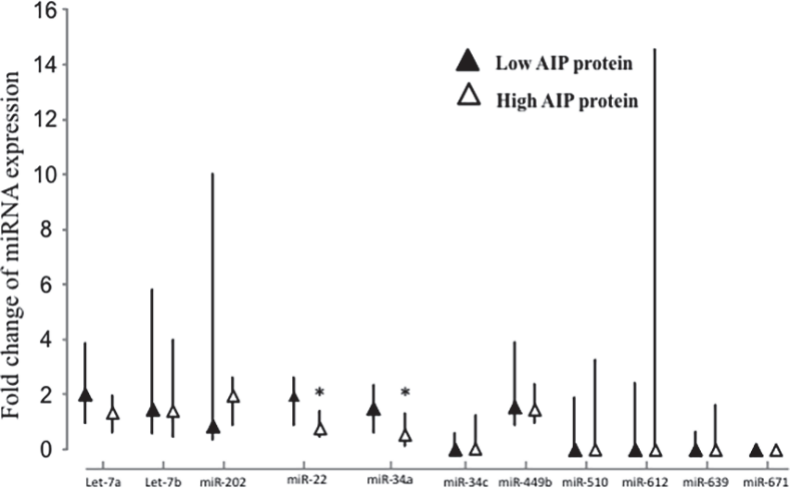
Expression levels of miRNAs predicted to bind *AIP* as determined by quantitative RT-PCR in human somatotroph adenomas with low (black triangles) and high (white triangles) AIP protein levels. Results are expressed as fold change compared to normal pituitary samples and are shown as median with upper and lower quartile; *, *P*<0.05.

Based on *in silico* predictions, we selected 11 miRNAs for analysis by real-time qPCR: let-7a, let-7b, miR-202, miR-22, miR-34a, miR-34c, miR-449b, miR-510, miR-612, miR-639 and miR-671 (Table 3). Two miRNAs showed higher expression in tumors with low AIP protein levels compared to tumors with high AIP protein levels (Fig. 2): miR-22 [fold change compared to normal pituitary, low AIP protein 1.97 (range 0.25–6.89) vs. high AIP protein 0.78 (range 0.13–9.78), *P* = 0.046] and miR-34a [low AIP protein 1.50 (range 0.28–3.37) vs. high AIP protein 0.55 (range 0.08–4.96), *P* = 0.022]. Nine out of 13 tumors (69%) with low AIP expression exhibited high miR-34a levels [i.e. higher than median (0.72) of the whole group]. There was no difference in the expression of the other miRNAs between tumors with low or high AIP protein levels (Fig. 2).

**Table 3:** The 11 miRNAs selected to analyse the expression level in patients with low or high AIP protein expression and the criteria for selection.

miRNA	Criteria for selection
Let-7a	Reachs all three miRNAmap criteria
Let-7b	Reachs all three miRNAmap criteria
miR-202	Two miRNAmap criteria and Pearson coefficient of-0.32
miR-22	Two miRNAmap criteria and Pearson coefficient of-0.33
miR-34a	Two miRNAmap criteria and Pearson coefficient of-0.31
miR-34c	Two miRNAmap criteria and Pearson coefficient of-0.43
miR-449b	Reachs all three miRNAmap criteria
miR-510	Two miRNAmap criteria and Pearson coefficient of-0.50
miR-612	Reachs all three miRNAmap criteria
miR-639	Reachs all three miRNAmap criteria
miR-671	Reachs all three miRNAmap criteria

### Correlation of AIP expression, miR-22 and miR-34a levels with tumor invasiveness, granulation pattern and response to somatostatin analogues

Eleven out of 13 (85%) somatotropinomas with low AIP protein expression were invasive while 6 out of 18 (33%) somatotropinomas with high AIP expression were invasive (*P =* 0.006) [Table 2]. The miR-34a levels were higher in invasive (1.30, range 0.12–3.37) than in non-invasive somatotropinomas (0.47, range 0.08–4.96); however, this difference was not statistically significant (*P* = 0.19).

Cytokeratin pattern was analyzed in 25 tumors. Eleven out of 25 somatotropinomas (44%) were classified as sparsely granulated. Nine (82%) sparsely granulated adenomas were invasive while only five (36%) densely granulated adenomas were considered invasive (*P* = 0.027). Eight out of 11 (73%) tumors with low AIP expression are sparsely granulated adenomas while only three out of 14 (21%) adenomas that presented a high AIP expression are sparsely granulated (*P* = 0.015). The miR-34a levels were 1.50 (range, 0.28–3.37) in sparsely granulated adenomas and 0.55 (0.08–4.96) in densely granulated adenomas, with a tendency to reach statistical difference (*P* = 0.058).

A total of 26 patients were initiated on OCT-LAR after surgery. In 10 patients (39%) acromegaly was considered controlled after OCT-LAR therapy. Only one out of nine patients (11%) whose tumors presented low AIP expression achieved disease control with medical treatment, while nine out of 17 patients (53%) harboring tumors with high AIP expression achieved disease control with OCT-LAR therapy (*P* = 0.045). The miR-34a levels were lower in those patients controlled with OCT-LAR therapy than in the uncontrolled patients [0.14 (range 0.08–4.96) and 1.12 (range 0.72–2.19), respectively, *P* = 0.003].

There was no correlation of miR-22 levels with tumor invasiveness, granulation pattern or response to OCT-LAR therapy.

### miR-34a and miR-22 predicted binding sites in the human *AIP*-3’UTR

**Fig 3:**
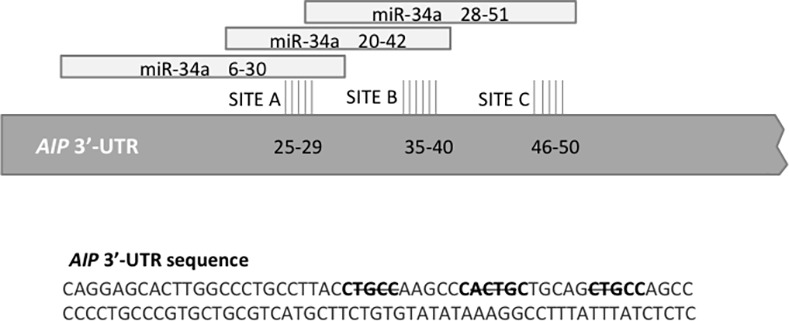
Graphic representation of the three predicted target sites of miR-34a in *AIP*-3’UTR (untranslated region). The highlighted sequences represent the three predicted binding sites (SITE A, B and C) and the basepairs marked with strikeout represent the deleted nucleotides in MUT_A, MUT_B and MUT_C plasmids.

FindTar predicted three different target seed regions for miR-34a in the human *AIP*-3’UTR sequence: site A, site B and site C, located respectively at 25–29 bp, 35–40 bp and 46–50 bp downstream of the stop codon of *AIP* (Fig. 3). Site B was predicted also by Microcosm. miRanda and FindTar predicted miR-34a to bind to the rat *Aip*-3’UTR sequence as well. miRanda predicted that miR-22 has one binding site, located 42–47 bp downstream of the stop codon of human *AIP*.

### miR-34a effect on regulation of AIP expression *in vitro*

**Fig 4:**
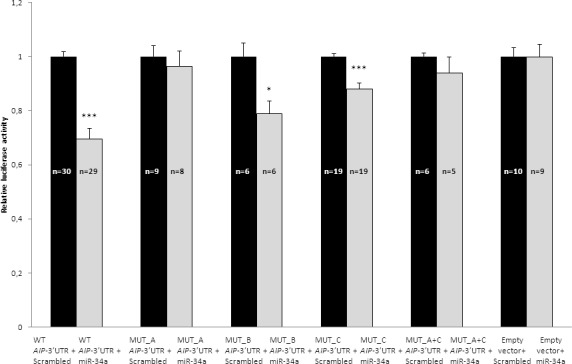
The effect of miR-34a on *AIP*-3’UTR activity in vitro. GH3 cells were transfected with plasmids containing pGL3-*AIP*-3’UTR WT and constructs with mutation in SITE A, SITE B, SITE C and SITE A+C and co-transfected with miR-34a or scrambled control. GH3 cells transfected with empty pGL3 vector and miR-34a or its scrambled control. Data are shown as Firefly/Renilla activity ratios compared to that of the scrambled control transfected cells. Mean ±SEM, *, *P*<0.05, ***, *P*<0.001.

**Fig 5:**
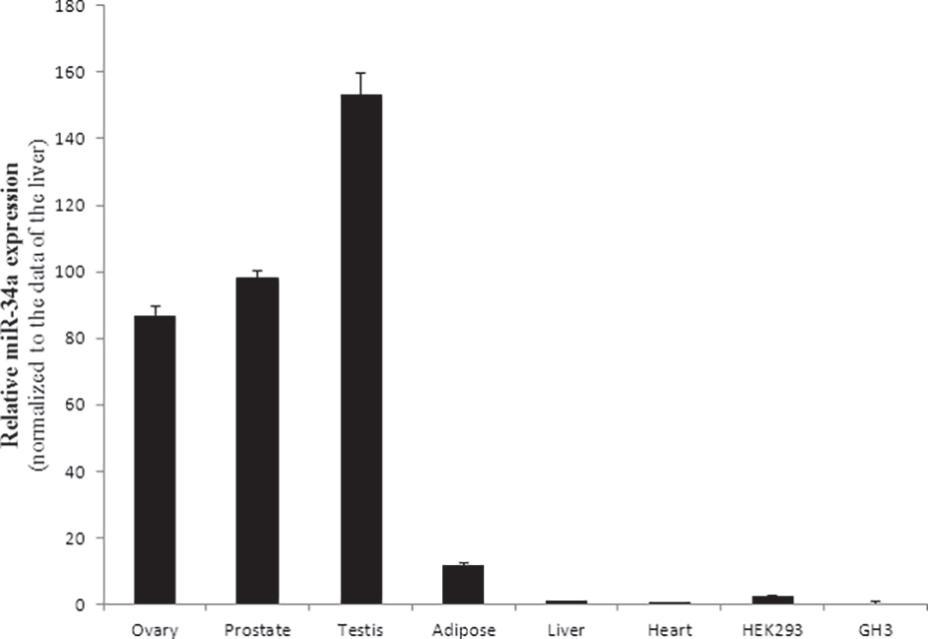
Endogenous miR-34a expression levels in different tissues and cell lines assessed in triplicates by RT-qPCR. Data were normalized to the data of the liver and shown as mean ±SEM.

To verify the *in silico* predicted interaction between miR-34a and miR-22 and *AIP*, we used a pGL3 vector containing the human wild type (WT) *AIP*-3’UTR downstream of the coding sequence of Firefly luciferase. Transfection of pre-miR-34a precursor and WT-*AIP*-3’UTR into GH3 cells resulted in a 31±4% reduction of luciferase activity compared with the control scrambled miR (*P*<0.0001) (Fig. 4). To confirm that this effect was caused by miR-34a interaction with the cloned fragment and not by nonspecific binding, we compared the effect exerted by the pre-miR-34a precursor and the scrambled miR on the empty pGL3 vector. miR-34a did not change the luciferase activity of the empty vector compared with the control scrambled miR (Fig. 4). As the endogenous level of miR-34a in GH3 cells was low (Fig. 5), we predict that the endogenous miR-34a did not interfere significantly in our experimental setting. Transfection of pre-miR-22 precursor and WT- *AIP*-3’UTR into GH3 cells resulted in no reduction of the luciferase activity (data not shown).

### Confirmation of predicted miR-34a binding sites

To confirm the importance of miRNA binding and to investigate which predicted binding site of miR-34a is involved in the miR-34a effect we used deletion mutants targeting the three different bindig sites: MUT_A for the mutated binding site A, MUT_B for site B and MUT_C for site C. MUT_A leads to a complete loss of miR-34a effect on luciferase activity (Fig. 4), while MUT_B did not change the inhibitory effect of miR-34a on the luciferase assay (Fig. 4). Although MUT_C overall did not change significantly the inhibitory effect of miR-34a, in some of the experiments a small effect was observed. Therefore we created a combined mutant of site A and site C: MUT_A+C to see if an additional effect of site A and C could be observed. The data with the combined mutant was similar to the one with MUT_A only (Fig. 4).

### Regulation of endogenous AIP expression by miR-34a *in vitro*

**Fig 6:**
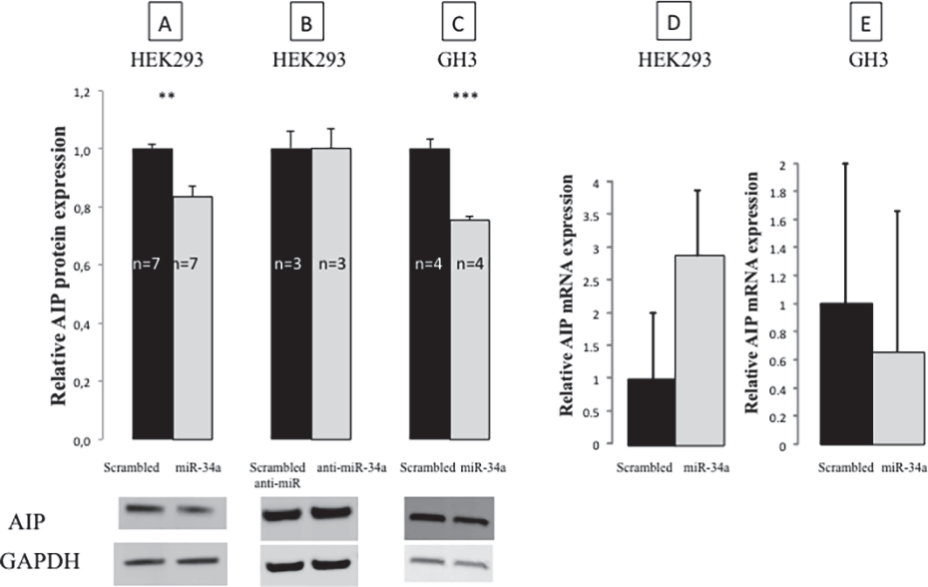
Effect of miR-34a (A) and anti-miR-34a (B) on endogenous AIP protein levels in HEK293 and in GH3 (C) cells 48 hours after transfection. Effect of miR-34a on endogenous AIP mRNA expression in HEK293 (D) and in GH3 (E) cells 48 hours after transfection measured by RT-qPCR. Data are shown as mean±SEM, **, *P*<0.01 ***, *P*<0.001.

To further characterize the interaction of miR-34a and *AIP in vitro* we measured mRNA and protein levels of endogenous AIP after miR-34a overexpression and inhibition in HEK293 cells. Significant decrease in AIP protein level was observed 48h post-transfection with miR-34a compared to scrambled miR control (n = 7, 17±3%, *P* = 0.001, Fig. 6A), suggesting that high levels of miR-34a can suppress endogenous AIP protein expression *in vitro*. Transfection with anti-miR-34a did not change AIP protein levels (n = 3, 0±7%, *P* = 0.998, Fig. 6B). We have also observed a significant decrease in endogenous AIP protein levels in GH3 cells after miR-34a overexpression (n = 4, 25±1%, *P* = 0.0005, Fig. 6C).

Although miR-34a overexpression induced a significant decrease in endogenous AIP protein levels, no significant change was seen at the mRNA level in HEK293 and GH3 cells, matching observations in our human adenomas. After miR-34a overexpression in HEK293 and GH3 cells we have observed no significant change in AIP mRNA levels compared to scrambled miR control (Fig. 6D, 6E).

## Discussion

In this study we showed that low AIP protein levels in human sporadic somatotropinomas is associated with high miR-34a expression and that miR-34a can down-regulate AIP protein levels in *in vitro* experiments. In addition, we showed that high miR-34a levels are associated with a lower chance of acromegaly control with SSA therapy and we confirmed our previous findings [7] that low AIP protein expression is associated with a poor response to SSA. Our data demonstrates that the inhibition involves AIP translation repression without reduction in AIP mRNA, as there was no difference in the AIP mRNA levels between tumors with low or high AIP protein levels.

Thirteen out of 31 (42%) tumor samples showed low AIP protein levels, a percentage similar to the data previously published by Jaffrain-Rea et al. (48%) [9] and also by our group in two different sets of samples (55% and 51%) [7, 8]. Most of the tumors exhibiting low AIP expression (with or without germline *AIP* mutations) were invasive [8, 9], and patients harboring those tumors have a poor response to the medical treatment with SSAs, the mainstay of the medical treatment of acromegaly [7]. Recent data suggest that the expression of AIP is important in the mechanism of action of this class of drugs [3, 17]. In addition, we showed that the majority of the tumors showing low AIP expression are sparsely granulated adenomas. Tumors with this cytokeratin pattern are known to be more invasive and associated with a poor response to SSA therapy [33]. Therefore, one of the possible explanations for this adenoma phenotype is the low AIP expression in these tumors.

As our data showed that there is no correlation between AIP mRNA expression and protein levels, we hypothesized that the low AIP protein levels could be explained by miRNA regulation. Therefore, we studied the miRNAs predicted to regulate the *AIP* gene. After a careful selection using *in silico* prediction, we studied 11 miRNAs. Although miR-107 has been previously shown to inhibit *AIP in vitro* [27], this miRNA did not fulfill the strict selection criteria in this study and was not included in the analysis. Out of our selected 11 miRNAs, two (miR-22 and miR-34a) were significantly overexpressed in the tumors with low AIP protein levels. We note that our sample size is the largest to date of the studies concentrating on miRNA expression in somatotropinomas. According to our results, we hypothesized that these miRNAs could be responsible for the reduced AIP levels. Our findings showed that miR-34a down-regulates AIP protein levels, while miR-22 had no inhibitory effect. We also showed that higher miR-34a levels are associated with a lower chance of disease control with SSA therapy. Therefore, elucidation of the mechanism involved in reducing AIP protein levels in sporadic somatotropinomas may help to predict response to SSAs and help with the development of novel therapeutic options for the treatment of this subgroup of invasive tumors.

To study the effect of miR-34a on AIP we have used a reporter vector in which the entire human *AIP*-3’-UTR has been fused to a luciferase reporter plasmid. We observed that high levels of miR-34a reduce the luciferase activity using the vector containing the WT *AIP*-3’UTR suggesting that miR-34a can bind to the *AIP*-3’UTR. To validate the three predicted target sites for miR-34a within the 3’-UTR of human *AIP* we created mutants for each predicted miR-34a binding sites. MUT_B and MUT_C plasmids exerted a significant inhibitory effect on the luciferase activity, similar to the wild-type plasmid, suggesting that these sites are not important for miR-34a binding. SITE_A plasmid, however, failed to induce inhibition of luciferase activity, suggesting that this binding site is responsible for miR-34a-mediated AIP repression. To study the regulation of endogenous AIP expression by miR-34a *in vitro* we have used rat GH3 cells and human HEK293 cells. We found that overexpression of miR-34a significantly decreases AIP protein levels measured 48h after transfection, while there was no significant change in AIP mRNA levels. When we transfected the cells with anti-miR-34a we could not see any change in AIP protein levels, which might be due to the fact that these cell lines have very low endogenous miR-34a levels therefore antagonism with anti-mir-34a may not produce detectable effects (Fig. 5). We have attempted to see if cells would proliferate more when transfected with miR-34a, and although a trend was observed, this has not reached significance (data not shown).

The miR-34 family consists of three miRNAs: miR-34a, miR34b and miR-34c. miR-34b and -34c share a common primary transcript (located on chromosome 11q23), while miR-34a is encoded by its own transcript on chromosome 1p26 [34]. miR-34a is an intergenic miRNA located between the genes coding for the G-protein coupled receptor 157 and hexose-6-phosphatase dehydrogenase [34]. Recent miRNA profiling analysis did not find differences in overall miR-34a expression in somatotropinomas compared to normal pituitary [11, 35]. This is in line with our findings that showed that miR-34a was overexpressed in tumors with low AIP expression, but not in all somatotropinomas. There were a few cases with high miR-34a level and high AIP expression. We have not identified sequence variants in the 3’UTR of the *AIP* gene in these samples. Therefore as high miR-34 expression does not always correlate with a low AIP protein level, other factors could also influence AIP expression. To elucidate the complex regulation of AIP expression will need future studies.

miRNAs have unique tissue-specific patterns and the same miRNA can behave as an oncogene or tumor suppressor gene depending on their target mRNAs in that particular organ [13, 36]. miR-34a has been shown to behave as a tumor suppressor miRNA by reducing proliferation and enhancing apoptosis in many human neoplasias, including osteosarcoma, colorectal, pancreatic and ovarian cancer [37, 38]. In contrast, miR-34a has also been shown to have oncomiR properties in other studies, including a pro-proliferative role in follicular lymphoma cell lines and an anti-apoptotic effect in B-lymphoid cells [39, 40]. It has also been shown to antagonize the anti-tumoral effects of docetaxel in human breast cancer cells [41]. Our data show higher miR-34a levels in human adenomas with low AIP protein levels. In addition, we demonstrated that miR-34a can bind to *AIP* and decrease its expression *in vitro*. Interestingly, miR-34a overexpression in HCT116 human colon carcinoma cells also showed down-regulation of AIP expression [42] (http://www.ncbi.nlm.nih.gov/geoprofiles/39833130), suggesting that the putative role of miR-34a in regulating AIP expression may also be present in other tumors. Patients with loss-of-function *AIP* mutations usually harbor large and invasive somatotropinomas [4, 5]. In the absence of mutations, low AIP protein level is associated with a similar phenotype [8, 9]. In this study we postulated that miR-34a acts as an oncomiR in somatotropinomas, being an inhibitor of *AIP*, a tumor suppressor gene, and therefore miR-34a might be implicated in the pathogenesis of these tumors. On the other hand, as miR-34 is involved in a wide range of tumorigenesis, the role of miR-34 on somatotropinomas may not solely rely on AIP.

The involvement of miR-34a in the pathogenesis of sporadic somatotropinomas may allow the development of new therapeutic strategies for the treatment of these tumors. The therapeutic inhibition of miR-34 has previously been attempted in the context of heart disease. This resulted in the attenuation of pathological cardiac remodeling and improvement in heart function in a mouse myocardial infarct model [43]. In the same study, the authors used a 15-mer locked nucleic acid (LNA) anti-miR-34a and observed that a single dose in three consecutive days inhibited the miR-34a as early as day one and that the inhibition persisted for two months after the last dose [43]. Therefore, future studies addressing the use of LNA anti-miR-34a in the setting of invasive somatotropinomas with low AIP protein levels may provide a new approach for the treatment of these tumors. The use of anti-miRNAs has been previously described in other tumor cell lines, for example, in an orthotopic xenograft breast cancer model with systemically injected liposomes that delivered 2’-O-Me anti-miRNAs against miR-132, resulting in delayed tumor growth and suppressed angiogenesis [44].

In conclusion, we have demonstrated that miR-34a is overexpressed in sporadic somatotropinomas with low AIP protein levels in the absence of mutations in this gene and that this overexpression is inversely correlated to the response to SSA. Functional studies confirmed that miR-34a down-regulates AIP expression, suggesting the possible involvement of miR-34a in the pathogenesis of sporadic somatotropinomas.

## References

[1] Vierimaa O, Georgitsi M, Lehtonen R, Vahteristo P, Kokko A, et al (2006) Pituitary adenoma predisposition caused by germline mutations in the AIP gene. Science 312: 1228–1230. 16728643 10.1126/science.1126100

[2] Beckers A, Aaltonen LA, Daly AF, Karhu A (2013) Familial isolated pituitary adenomas (FIPA) and the pituitary adenoma predisposition due to mutations in the aryl hydrocarbon receptor interacting protein (AIP) gene. Endocr Rev 34: 239–277. 10.1210/er.2012-1013 23371967 PMC3610678

[3] Gadelha MR, Kasuki L, Korbonits M (2013) Novel pathway for somatostatin analogs in patients with acromegaly. Trends Endocrinol Metab 24: 238–246. 10.1016/j.tem.2012.11.007 23270713

[4] Leontiou CA, Gueorguiev M, van der Spuy J, Quinton R, Lolli F, et al (2008) The role of the aryl hydrocarbon receptor-interacting protein gene in familial and sporadic pituitary adenomas. J Clin Endocrinol Metab 93: 2390–2401. 10.1210/jc.2007-2611 18381572

[5] Daly AF, Tichomirowa MA, Petrossians P, Heliovaara E, Jaffrain-Rea ML, et al (2010) Clinical characteristics and therapeutic responses in patients with germ-line AIP mutations and pituitary adenomas: an international collaborative study. J Clin Endocrinol Metab 95: E373–383. 10.1210/jc.2009-2556 20685857

[6] Raitila A, Georgitsi M, Karhu A, Tuppurainen K, Makinen MJ, et al (2007) No evidence of somatic aryl hydrocarbon receptor interacting protein mutations in sporadic endocrine neoplasia. Endocr Relat Cancer 14: 901–906. 17914118 10.1677/ERC-07-0025

[7] Kasuki L, Vieira Neto L, Wildemberg LE, Colli LM, de Castro M, et al (2012) AIP expression in sporadic somatotropinomas is a predictor of the response to octreotide LAR therapy independent of SSTR2 expression. Endocr Relat Cancer 19: L25–29. 10.1530/ERC-12-0020 22420004

[8] Kasuki Jomori de Pinho L, Vieira Neto L, Armondi Wildemberg LE, Gasparetto EL, Marcondes J, et al (2011) Low aryl hydrocarbon receptor-interacting protein expression is a better marker of invasiveness in somatotropinomas than Ki-67 and p53. Neuroendocrinology 94: 39–48. 10.1159/000322787 21178332

[9] Jaffrain-Rea ML, Angelini M, Gargano D, Tichomirowa MA, Daly AF, et al (2009) Expression of aryl hydrocarbon receptor (AHR) and AHR-interacting protein in pituitary adenomas: pathological and clinical implications. Endocr Relat Cancer 16: 1029–1043. 10.1677/ERC-09-0094 19556287

[10] Palumbo T, Faucz FR, Azevedo M, Xekouki P, Iliopoulos D, et al (2013) Functional screen analysis reveals miR-26b and miR-128 as central regulators of pituitary somatomammotrophic tumor growth through activation of the PTEN-AKT pathway. Oncogene 32: 1651–1659. 10.1038/onc.2012.190 22614013 PMC4034118

[11] D'Angelo D, Palmieri D, Mussnich P, Roche M, Wierinckx A, et al (2012) Altered microRNA expression profile in human pituitary GH adenomas: down-regulation of miRNA targeting HMGA1, HMGA2, and E2F1. J Clin Endocrinol Metab 97: E1128–1138. 10.1210/jc.2011-3482 22564666

[12] Bottoni A, Zatelli MC, Ferracin M, Tagliati F, Piccin D, et al (2007) Identification of differentially expressed microRNAs by microarray: a possible role for microRNA genes in pituitary adenomas. J Cell Physiol 210: 370–377. 17111382 10.1002/jcp.20832

[13] Carthew RW, Sontheimer EJ (2009) Origins and Mechanisms of miRNAs and siRNAs. Cell 136: 642–655. 10.1016/j.cell.2009.01.035 19239886 PMC2675692

[14] Farazi TA, Spitzer JI, Morozov P, Tuschl T (2011) miRNAs in human cancer. J Pathol 223: 102–115. 10.1002/path.2806 21125669 PMC3069496

[15] Giustina A, Chanson P, Bronstein MD, Klibanski A, Lamberts S, et al (2010) A consensus on criteria for cure of acromegaly. J Clin Endocrinol Metab 95: 3141–3148. 10.1210/jc.2009-2670 20410227

[16] Vieira Neto L, Abucham J, Araujo LA, Boguszewski CL, Bronstein MD, et al (2011) [Recommendations of Neuroendocrinology Department from Brazilian Society of Endocrinology and Metabolism for diagnosis and treatment of acromegaly in Brazil]. Arq Bras Endocrinol Metabol 55: 91–105. 10.1590/S0004-27302011000200001 21584426

[17] Chahal HS, Trivellin G, Leontiou CA, Alband N, Fowkes RC, et al (2012) Somatostatin analogs modulate AIP in somatotroph adenomas: the role of the ZAC1 pathway. J Clin Endocrinol Metab 97: E1411–1420. 10.1210/jc.2012-1111 22659247

[18] Knosp E, Steiner E, Kitz K, Matula C (1993) Pituitary adenomas with invasion of the cavernous sinus space: a magnetic resonance imaging classification compared with surgical findings. Neurosurgery 33: 610–617. 8232800 10.1227/00006123-199310000-00008

[19] Kasuki L, Wildemberg LE, Neto LV, Marcondes J, Takiya CM, et al (2013) Ki-67 is a predictor of acromegaly control with octreotide LAR independent of SSTR2 status and relates to cytokeratin pattern. Eur J Endocrinol 169: 217–223. 10.1530/EJE-13-0349 23749849

[20] Obari A, Sano T, Ohyama K, Kudo E, Qian ZR, et al (2008) Clinicopathological features of growth hormone-producing pituitary adenomas: difference among various types defined by cytokeratin distribution pattern including a transitional form. Endocr Pathol 19: 82–91. 10.1007/s12022-008-9029-z 18629656

[21] Pabinger S, Thallinger GG, Snajder R, Eichhorn H, Rader R, et al (2009) QPCR: Application for real-time PCR data management and analysis. BMC Bioinformatics 10: 268 10.1186/1471-2105-10-268 19712446 PMC2741456

[22] Hsu SD, Chu CH, Tsou AP, Chen SJ, Chen HC, et al (2008) miRNAMap 2.0: genomic maps of microRNAs in metazoan genomes. Nucleic Acids Res 36: D165–169. 18029362 10.1093/nar/gkm1012 PMC2238982

[23] Lu J, Getz G, Miska EA, Alvarez-Saavedra E, Lamb J, et al (2005) MicroRNA expression profiles classify human cancers. Nature 435: 834–838. 15944708 10.1038/nature03702

[24] Bancroft FC, Levine L, Tashjian AH Jr. (1969) Control of growth hormone production by a clonal strain of rat pituitary cells. Stimulation by hydrocortisone. J Cell Biol 43: 432–441. 5389137 10.1083/jcb.43.3.432 PMC2107805

[25] Tashjian AH Jr., Yasumura Y, Levine L, Sato GH, Parker ML (1968) Establishment of clonal strains of rat pituitary tumor cells that secrete growth hormone. Endocrinology 82: 342–352. 4951281 10.1210/endo-82-2-342

[26] Graham FL, Smiley J, Russell WC, Nairn R (1977) Characteristics of a human cell line transformed by DNA from human adenovirus type 5. J Gen Virol 36: 59–74. 886304 10.1099/0022-1317-36-1-59

[27] Trivellin G, Butz H, Delhove J, Igreja S, Chahal HS, et al (2012) MicroRNA miR-107 is overexpressed in pituitary adenomas and in vitro inhibits the expression of aryl hydrocarbon receptor-interacting protein (AIP). Am J Physiol Endocrinol Metab 303: E708–719. 10.1152/ajpendo.00546.2011 22811466

[28] Patel MI, Tuckerman R, Dong Q (2005) A Pitfall of the 3-(4,5-dimethylthiazol-2-yl)-5(3-carboxymethonyphenol)-2-(4-sulfophenyl)-2H-tetra zolium (MTS) assay due to evaporation in wells on the edge of a 96 well plate. Biotechnol Lett 27: 805–808. 16086264 10.1007/s10529-005-5803-x

[29] Nanzer AM, Khalaf S, Mozid AM, Fowkes RC, Patel MV, et al (2004) Ghrelin exerts a proliferative effect on a rat pituitary somatotroph cell line via the mitogen-activated protein kinase pathway. Eur J Endocrinol 151: 233–240. 15296479 10.1530/eje.0.1510233

[30] Daly AF, Vanbellinghen JF, Khoo SK, Jaffrain-Rea ML, Naves LA, et al (2007) Aryl hydrocarbon receptor-interacting protein gene mutations in familial isolated pituitary adenomas: analysis in 73 families. J Clin Endocrinol Metab 92: 1891–1896. 17244780 10.1210/jc.2006-2513

[31] Igreja S, Chahal HS, King P, Bolger GB, Srirangalingam U, et al (2010) Characterization of aryl hydrocarbon receptor interacting protein (AIP) mutations in familial isolated pituitary adenoma families. Hum Mutat 31: 950–960. 10.1002/humu.21292 20506337 PMC3065644

[32] Zatelli MC, Torre ML, Rossi R, Ragonese M, Trimarchi F, et al (2013) Should aip gene screening be recommended in family members of FIPA patients with R16H variant? Pituitary 16: 238–244. 10.1007/s11102-012-0409-5 22915287

[33] Fougner SL, Casar-Borota O, Heck A, Berg JP, Bollerslev J (2012) Adenoma granulation pattern correlates to clinical variables and effect of somatostatin analogue treatment in a large series of patients with acromegaly. Clin Endocrinol (Oxf) 76: 96–102. 10.1111/j.1365-2265.2011.04163.x 21722151

[34] Hermeking H (2010) The miR-34 family in cancer and apoptosis. Cell Death Differ 17: 193–199. 10.1038/cdd.2009.56 19461653

[35] Mao ZG, He DS, Zhou J, Yao B, Xiao WW, et al (2010) Differential expression of microRNAs in GH-secreting pituitary adenomas. Diagn Pathol 5: 79 10.1186/1746-1596-5-79 21138567 PMC3017030

[36] Fabian MR, Sonenberg N, Filipowicz W (2010) Regulation of mRNA translation and stability by microRNAs. Annu Rev Biochem 79: 351–379. 10.1146/annurev-biochem-060308-103103 20533884

[37] Vogt M, Munding J, Gruner M, Liffers ST, Verdoodt B, et al (2011) Frequent concomitant inactivation of miR-34a and miR-34b/c by CpG methylation in colorectal, pancreatic, mammary, ovarian, urothelial, and renal cell carcinomas and soft tissue sarcomas. Virchows Arch 458: 313–322. 10.1007/s00428-010-1030-5 21225432

[38] Yan K, Gao J, Yang T, Ma Q, Qiu X, et al (2012) MicroRNA-34a inhibits the proliferation and metastasis of osteosarcoma cells both in vitro and in vivo. PLoS One 7: e33778 10.1371/journal.pone.0033778 22457788 PMC3310405

[39] Rizzo M, Mariani L, Cavallini S, Simili M, Rainaldi G (2012) The over-expression of miR-34a fails to block DoHH2 lymphoma cell proliferation by reducing p53 via c-MYC down-regulation. Nucleic Acid Ther 22: 283–288. 10.1089/nat.2012.0343 22830357

[40] Sotillo E, Laver T, Mellert H, Schelter JM, Cleary MA, et al (2011) Myc overexpression brings out unexpected antiapoptotic effects of miR-34a. Oncogene 30: 2587–2594. 10.1038/onc.2010.634 21297663 PMC3128883

[41] Kastl L, Brown I, Schofield AC (2012) miRNA-34a is associated with docetaxel resistance in human breast cancer cells. Breast Cancer Res Treat 131: 445–454. 10.1007/s10549-011-1424-3 21399894

[42] Chang TC, Wentzel EA, Kent OA, Ramachandran K, Mullendore M, et al (2007) Transactivation of miR-34a by p53 broadly influences gene expression and promotes apoptosis. Mol Cell 26: 745–752. 17540599 10.1016/j.molcel.2007.05.010 PMC1939978

[43] Bernardo BC, Gao XM, Winbanks CE, Boey EJ, Tham YK, et al (2012) Therapeutic inhibition of the miR-34 family attenuates pathological cardiac remodeling and improves heart function. Proc Natl Acad Sci U S A 109: 17615–17620. 10.1073/pnas.1206432109 23047694 PMC3491509

[44] Anand S, Majeti BK, Acevedo LM, Murphy EA, Mukthavaram R, et al (2010) MicroRNA-132-mediated loss of p120RasGAP activates the endothelium to facilitate pathological angiogenesis. Nat Med 16: 909–914. 10.1038/nm.2186 20676106 PMC3094020

